# Comparison of the biometric values obtained by two different A-mode ultrasound devices (Eye Cubed vs. PalmScan): A Transversal, descriptive, and comparative study

**DOI:** 10.1186/1471-2415-10-8

**Published:** 2010-03-24

**Authors:** Raul Velez-Montoya, Eugene Mark Shusterman, Miriam Jessica López-Miranda, Mariana Mayorquin-Ruiz, Guillermo Salcedo-Villanueva, Hugo Quiroz-Mercado, Virgilio Morales-Cantón

**Affiliations:** 1 Retina Department, Asociación para Evitar la Ceguera en México, 46 Vicente García Torres, Mexico DF 04030, Mexico; 2 Medical Director. Oraya Therapeutics, Inc. 8000 Jarvis Ave. Newark, CA 94560, USA; 3 Ocular Image Department, Asociación para Evitar la Ceguera en México, 46 Vicente García Torres, Mexico DF 04030, Mexico; 4 Ophthalmology Department, Denver Health Medical Center. 777 Bannock Street, Denver, CO 80204, USA

## Abstract

**Background:**

To assess the reliability of the measurements obtained with the PalmScan™, when compared with another standardized A-mode ultrasound device, and assess the consistency and correlation between the two methods.

**Methods:**

Transversal, descriptive, and comparative study. We recorded the axial length (AL), anterior chamber depth (ACD) and lens thickness (LT) obtained with two A-mode ultrasounds (PalmScan™ A2000 and Eye Cubed™) using an immersion technique. We compared the measurements with a two-sample *t*-test. Agreement between the two devices was assessed with Bland-Altman plots and 95% limits of agreement.

**Results:**

70 eyes of 70 patients were enrolled in this study. The measurements with the Eye Cubed™ of AL and ACD were shorter than the measurements taken by the PalmScan™. The differences were not statistically significant regarding AL (p < 0.4) but significant regarding ACD (p < 0.001). The highest agreement between the two devices was obtained during LT measurement. The PalmScan™ measurements were shorter, but not statistically significantly (p < 0.2).

**Conclusions:**

The values of AL and LT, obtained with both devices are not identical, but within the limits of agreement. The agreement is not affected by the magnitude of the ocular dimensions (but only between range of 20 mm to 27 mm of AL and 3.5 mm to 5.7 mm of LT). A correction of about 0.5 D could be considered if an intraocular lens is being calculated. However due to the large variability of the results, the authors recommend discretion in using this conversion factor, and to adjust the power of the intraocular lenses based upon the personal experience of the surgeon.

## Background

The accuracy of biometric assessments (axial length, anterior chamber depth, lens thickness and central pachymetry) is a point of extreme importance for the evaluation of eye pathology, particularly when planning lens replacement surgery or other therapeutic procedures [1-3]. Currently, the eye specialist has several options for making such measurements [2].

The physical principles underlying these devices are varied. The latest generation technology is based on the principle of partial coherence interferometry, which uses a light source to make measurements [1,4]. The most accessible technique remains the use of sound waves, emitted by a piezoelectric crystal and delivered with a probe ranging in frequency from 8 to 50 MHz (ultrasound). Determination of eye morphometry via ultrasound is based upon the differential return of the ultrasonic waves by varying tissue types [4].

A-mode ultrasound is performed using a 10 MHz probe, which allows a resolution of 200 μm and a longitudinal clinical accuracy of 100 to 200 μm [1,2]. The contact method has a higher probability of error because the measurements are significantly shorter, and more variable due to unpredictable corneal compression with the probe [1,5]. The immersion technique eliminates this source of error by removing probe contact, as it remains between 5 to 10 mm away from the cornea, allowing more precise measurements [4,6,7].

The PalmScan™ A2000 (Micro Medical Devices, Calabasas, CA) is a portable system, which supports A-mode immersion biometry. The aim of this study is to assess the reliability of the measurements obtained with the PalmScan™, when compared with another standardized A-mode ultrasound device (Eye Cubed™, Ellex, Adelaide, Australia) and assess the consistency and agreement between the two methods, in order to use the two systems interchangeably.

## Methods

A transversal, descriptive, and comparative study was conducted at the retina department of our hospital. The study was reviewed and approved by the hospital's bioethics and scientific research board. All procedures were performed according to the tenets of the declaration of Helsinki, and informed consent was obtained from all participants after a complete explanation about the nature of the study, and before any measurements were made.

The patients were randomly selected from the ambulatory service of our hospital, but eliminating patients with high myopia (-8 diopters or higher), corneal irregularities, history of retinal tumors, retinal fibrosis, retina surgery, active or chronic uveitis, glaucoma, recent eye surgery (less than three months from recruitment), currently active ocular or periocular infections, or those who were not willing to sign the informed consent.

We recorded the sex, age, and lens status (phakic or pseudophakic) of all patients. We measured only the right eye of each patient, first with the Eye Cubed™ device and a few minutes later with the PalmScan™. The measured biometric parameters were the axial length (AL), defined as the distance between the anterior corneal surface and the internal limiting membrane, the anterior chamber depth (ACD), defined as the distance between the distance between anterior corneal surface and the anterior lens capsule, and the lens thickness (LT), defined as the space between the anterior and posterior lens capsule. All scans were performed by the same experienced investigator. Each eye was measured 10 times with both ultrasound devices using an immersion technique with a 10 MHz probe. The average speed for the conversion of milliseconds to millimeters was 1532 m/s for the vitreous and anterior chamber, and 1641 m/s for the lens.

The measuring technique was as follows:

### Eye Cubed™

With the corneal surface properly anesthetized using 0.25% tetracaine drops, and the patient in supine position, an immersion shell was placed on the eye surface and filled with balanced saline solution. Then the patient was asked to set the eyes on a distant point. The tip of the probe was submerged 3 to 4 mm in the saline solution but still suspended 5 to 8 mm away from the anterior corneal surface. The measurements were recorded directly from the device.

### PalmScan™ A2000

**Figure 1:**
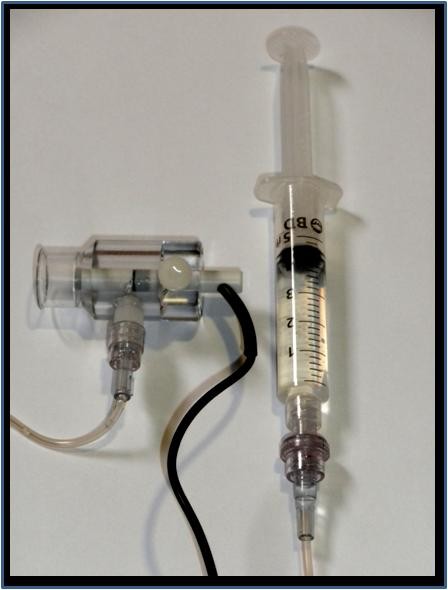
**Immersion probe and PalmScan™ device main display**.

With this device, we used the special immersion probe provided by the manufacturer (Figure 1). The cornea was anesthetized with 0.25% tetracaine drops. The probe was then placed over the limbus and filled with balanced saline solution to the immersion mark on the probe shell. The patients were then asked to fixate on the light emitted by the probe, as per manufacturer's instructions. The measurements were recorded directly from the device. Settings were appropriately adjusted to account for phakic vs. pseudophakic status, as per device manual.

Statistical analysis was performed using SPSS software, version 10.1.0 (Inc. SPSS Chicago, IL). After assessing the type of distribution of both groups with the proper histograms (which was a normal distribution; data not shown), the differences between LT, ACD and LT were explored using a two-sample *t*-test (with equal and unequal variance, as appropriate). A two-tailed *p *value of less than 0.05 was considered significant (0.95 level of confidence).

### Bland-Altman plots

The agreement of the three measurements (AL, ACD & LT) between the two devices was assessed with Bland-Altman plots and 95% limits of agreement. We plotted on the X axis the difference found between the PalmScan™ and the Eye Cubed™, against the average of the two measurements plotted in the Y axis. The mean and standard deviations were obtained using the SPSS software, version 10.1.0 (Inc. SPSS Chicago, IL), and then added to the plots manually.

## Results

We included 70 eyes of 70 patients (29 males and 41 females). All patients met the inclusion/exclusion criteria. The mean age was 54.71 ± 22.33 years. Sixty-four patients were phakic and six were pseudophakic. The mean biometric measurements of AL, ACD and LT are summarized in table 1. There were no statistical differences between the AL and LT in the phakic and pseudophakic measurements (*p *> 0.2). There was a significant difference between the measurements of ACD in the phakic patients (*p *< 0.0001). This difference was not sustained in the pseudophakic patients.

**Table 1:** Biometric Measurements.

*Phakic:*	Axial Length	Anterior Chamber Deep	Lens Thickness
**PalmScan:**	23.33 ± 0.97	3.25 ± 0.36	4.52 ± 0.55
**Eye Cubed:**	23.20 ± 0.97	2.73 ± 0.42	4.59 ± 0.55

*p*	0.4	<0.0001	0.7

*Pseudophakic:*			
**PalmScan:**	24.52 ± 1.42	4.37 ± 0.47	0.70 ± 0.00
**Eye Cubed:**	24.44 ± 1.60	3.99 ± 0.47	0.76 ± 0.11

*p*	0.93	0.1	0.2

Means ± standard deviation in mm. All the patients in the pseudophakic group obtained the same value of lens thickness with the PalmScan™. Due to this, and because there were only six patients in this group, the standard deviation equals zero.

**Figure 2:**
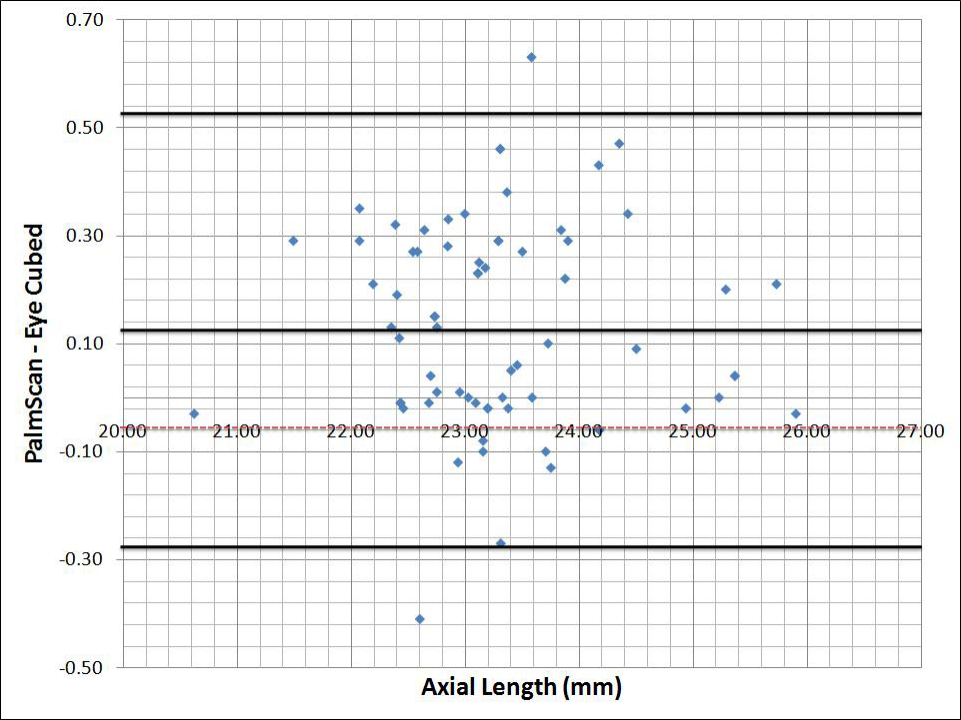
**Bland-Altman plot showing interdevice difference plotted against mean measurements for each eye**. Dotted line: zero line. Black solid line, mean difference and boundaries of the 95% limits of agreement.

**Figure 3:**
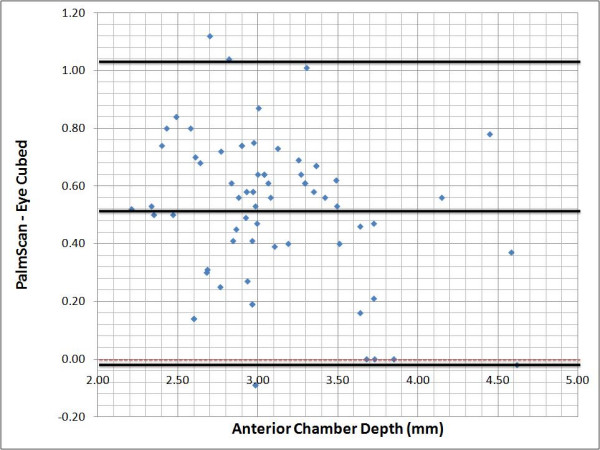
**Bland-Altman plot showing interdevice difference plotted against mean measurements for each eye**. Dotted line: zero line. Black solid line, mean difference and boundaries of the 95% limits of agreement. Strong positive trend, with a clinically significant alpha value (*p *< 0.0001). Bland-Altman plot showing interdevice difference plotted against mean measurements for each eye. Dotted line, zero line. Black solid line, mean difference and boundaries of the 95% limits of agreement.

The Bland-Altman plots show that the three measurements done with both devices are between the limit of agreement. Although the agreement is not perfect (differences ? 0), the range of the measurements falls within the respective standard deviations. In the case of the AL (Figure 2), the positive average of the differences (+0.13) indicates that the measurements taken with the Eye Cubed™ system are slightly shorter than those obtained with the PalmScan™; however, the agreement of the measurements is not affected by the magnitude of the AL. In the case of the ACD (Figure 3), the measurements obtained by the Eye Cubed™ are shorter again (mean +0.51). The difference between the two devices was significant (p < 0.0001). The correlation of the measurements was not affected by the magnitude of the ACD.

**Figure 4:**
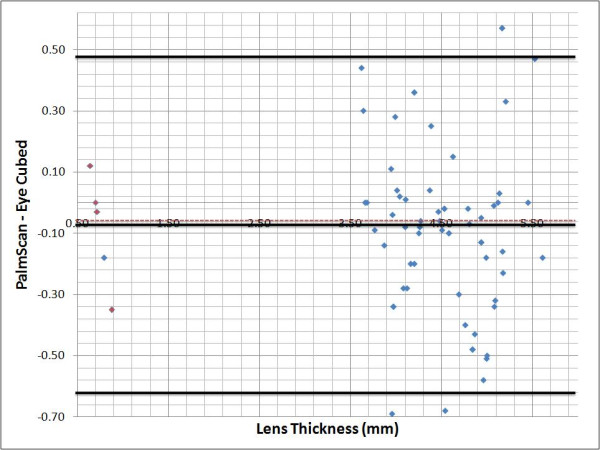
**Bland-Altman plot showing interdevice difference plotted against mean measurements for each eye**. Dotted line, zero line. Black solid line, mean difference and boundaries of the 95% limits of agreement. Red dots, pseudophakic group.

Finally, in the case of the LT, both devices showed the highest level of agreement between the measurements, with the trend of the differences toward zero (Figure 4), the average slightly negative (-0.08), indicating that the measurements made by the PalmScan™ were shorter. Again, the agreement between the measurements was not affected by the magnitude of the LT.

## Discussion

The results of this study have shown that, even thought the values of AL and LT obtained with both systems are not identical, the measurements are between the limits of agreement (± 1.96 standard deviations). The level of agreement is not affected by the magnitude of the ocular dimensions (but only the range of 20 to 27 mm of AL and 3.5 to 5.7 mm of LT). Therefore, the interchangeable use of the PalmScan™ device as a method of measurement of those variables is feasible.

Regarding the ACD, the difference between the measurements was statistically significant, with the values of the PalmScan™ much longer than those obtained by the Ellex Eye Cubed™. The authors speculate that this difference could be due to human error during the placement of the landmarks for measuring the ultrasound spikes with the Eye Cubed™, while in the PalmScan™ this placement was done automatically by the system. Another explanation could be that the measurements with the PalmScan™ were made after cyclopegic eye drops. However, as the study progressed, this latter factor was eliminated as a potential source of error.

The population involved in this study was Hispanic in origin. If we compare our AL values with those obtained in the Los Angeles Latino Eye Study (23.25 mm vs. 23.1 mm) we can see that our results are longer, being more similar to those reported in a study of Asian population, the Tanjong Pagar Survey (23.25 mm vs. 23.2 mm) [8,9]. As for the two ACD values obtained by the devices (Eye Cubed™: 2.73 mm and PalmScan™: 3.25), these are similar to those reported by Kriechbaum (2.87 mm) and Koranyi (3.05 mm) [10,11]. Also the Eye Cubed™ ACD value are very similar to those reported by Nemeth et al (2.95 mm ± 0.34 mm), which also used ultrasound with immersion technique, while the value obtained by the PalmScan™ is closer to the measurement obtained by the anterior segment optical coherence tomography (Visante, Zeiss, Meditech, Dublin, CA) which is 3.12 ± 0.33 mm [12,13].

Regarding intraocular lens power calculation, although the difference of the AL measurement between the two devices is only about 0.13 mm with no statistical difference between them, in practice this difference could represent a variation of the final refraction around 0.5 D. therefore this result should be taken with caution; a simple adjustment of the intraocular lens power with a correction factor of 0.5 D is not enough because of the large variability of the study. The proper adjustment should be made according to the surgeon's personal experience.

Finally, our study has some limitations that we would like to mention. First, the patients were chosen randomly, regardless of whether they were phakic or pseudophakic, or if the lens had some degree of opacity or whether patients were diabetic or not diabetic. Some of these factors might have caused some measurements to be extreme and out of the group standard deviation [14]. Another limitation was that we followed the protocol of our hospital ultrasound department, where traditionally the measurements of the A-mode ultrasound are made manually and not automatically. This may have caused the error in measuring the ACD, as mentioned previously.

## Conclusions

The PalmScan™ is a portable device, easy to handle, whose measurements of AL and LT are similar to those obtained by another A-mode ultrasound device, that despite such measurements are not identical, these are within an acceptable limit of agreement. Although the result of this study show a significant difference between the two systems for measuring ACD, the design of this study cannot clarify whether that differences was due to a human error or to the measurement methods. Finally, the similarity of the AL measurements between both devices tempts the user to try to apply a correction factor about 0.5 D while calculating intraocular lens power, but due to the large variability of this study results the authors recommend discretion and to adjust the power based upon the personal experience of each surgeon.

## Competing interests

None of the authors have a financial or proprietary interest in the publication of this paper or in the technology described. The authors declare that they have no competing interest in the publication of this paper.

## Authors' contributions

RVM: Conceived the study, participated in its design, made the patient selection, carried out the measurements, performed the statistical analysis, participated in the paper preparation, reviewed the manuscript and approved the final version. EMS: Conceived the study, participated in its design, participated in the paper preparation, reviewed and approved the final version of the paper, which includes English language revision. MJLM: Participated in the patient selection, carried out the measurements and participated in the paper preparation. GSV: Participated in the patient selection and carried out the informed consent of all patients. He also made the measurements of the control group and participated in the paper preparation. MMR: Carried out patient recruitment, patient selection and reviewed the measurements. She participated in the edition and approved the final manuscript.HQM: Participated in the paper preparation, paper review and approved the final version of the manuscript. VMC: Conceived the study and participated in the paper preparation, paper review and approved the final version of the manuscript.

All authors read and approved the final version of the manuscript, including the lattes version with the major and minor edition changes asked by the journal.

## Note

The paper has been partially presented at the Association for Research in Vision and Ophthalmology (ARVO) meeting 2009, Fort Lauderdale, Florida.

## Pre-publication history

The pre-publication history for this paper can be accessed here:

http://www.biomedcentral.com/1471-2415/10/8/prepub
